# Structural Analysis of Women’s Heptathlon

**DOI:** 10.3390/sports4010012

**Published:** 2016-02-19

**Authors:** Freya Gassmann, Michael Fröhlich, Eike Emrich

**Affiliations:** 1 Department of Economics and Sociology of Sport, Institute for Sport Science, Saarland University, Campus Gebäude B8 1, Saarbrücken 66123, Germany; e.emrich@mx.uni-saarland.de; 2 Department of Sports Science, University of Kaiserslautern, Erwin-Schrödinger-Street, Kaiserslautern 67663, Germany; michael.froehlich@sowi.uni-kl.de

**Keywords:** athletics, rule change, cluster analysis, factor analysis, competitive performance

## Abstract

The heptathlon comprises the results of seven single disciplines, assuming an equal influence from each discipline, depending on the measured performance. Data analysis was based on the data recorded for the individual performances of the 10 winning heptathletes in the World Athletics Championships from 1987 to 2013 and the Olympic Games from 1988 to 2012. In addition to descriptive analysis methods, correlations, bivariate and multivariate linear regressions, and panel data regressions were used. The transformation of the performances from seconds, centimeters, and meters into points showed that the individual disciplines do not equally affect the overall competition result. The currently valid conversion formula for the run, jump, and throw disciplines prefers the sprint and jump disciplines but penalizes the athletes performing in the 800 m run, javelin throw, and shotput disciplines. Furthermore, 21% to 48% of the variance of the sum of points can be attributed to the performances in the disciplines of long jump, 200 m sprint, 100 m hurdles, and high jump. To balance the effects of the single disciplines in the heptathlon, the formula to calculate points should be reevaluated.

## 1. Introduction and Theoretical Framework

The women’s heptathlon is an additive competition consisting of seven disciplines. Within two competition days, the women perform (in order) the disciplines of 100 m hurdles, high jump, shotput, and a 200 m run on the first day and a long jump, javelin throw, and 800 m run on the second day. The result in points achieved in the seven disciplines based on individual performances is determined by a points system that was changed last in 1985 [1,2]. The idea of the heptathlon implicitly assumes a relative balance of the single disciplines on the overall performance. Therefore, a conversion formula for the run, jump, and throw disciplines has been developed by Karl Ulbrich and applied for almost 30 years according to the International Association of Athletics Federations (IAAF). The conversion formula contains the following components:

(1) Run competitions (200 m, 800 m, and 100 m hurdles) = P = A × (B − T)^C^; T = time in seconds;
(2) Jump competitions (high jump and long jump) = P = A × (M − B)^C^; M = measurement result in centimeters;
(3) Throw competitions (shotput and javelin throw) = P = A × (D − B)^C^; D = distance in meters.

These components result in a power function with the constants A, B, and C with discipline-specific constants. In this context, Pitsch *et al.* [3] noted that the disciplines are not weighted equally. Increasing performance in some disciplines is reflected in only a slightly progressive increase in points.

The general problem in determining the sum of points achieved in the heptathlon is that, on the one hand, the evaluation of the individual performance in a discipline follows normative assumptions about the transformation rules, and on the other hand, linearity between performances measured and points achieved is impossible [3,4]. Letzelter [5] already noted this source of error by stating that the linear transformation of performances into points negates the "law of quantity" of training and denies the "principle of progressive load". Hence, normative assumptions per se influence the required equal weighting of individual disciplines [6] and contradict the principle of equally weighted balance between the disciplines, as formulated by Trkal [1] for example:

“ … that the new tables should be developed according to the following nine principles: … 3). The tables in all disciplines should be: a modification of current tables, linear in all disciplines, very slightly progressive in all disciplines […] 9) As far as possible, the tables should eliminate the possibility that an athlete specializing in one discipline is able to acquire sufficient points in that disciplines to overcome a low scores in weaker disciplines and beat more versatile all-round athletes.”

Concerning the general principles of balance as expressed explicitly in Principle 9, Kenny *et al.* [7], Westera [6] and others noted within the context of the decathlon that further analyses are required. In particular, the questions arise regarding the extent to which performances in single disciplines are included in the total result in an equally weighted form and the contribution of each discipline to the overall result. 

Regarding the single heptathlon disciplines, Westera [2] further notes the following:

“When starting from the principle of all roundness, the ideal score distribution should be uniform over the disciplines. The large deviations from uniformity prompt for a revision of the current scoring method.”

Therefore, analogous to the studies about the influence of a single discipline on the total result in the men's decathlon [8,9,10], the following questions are examined.

(1) The extent to which a specific discipline and/or cluster of disciplines determines the total number of points to a more than random high scale in the women’s heptathlon [2,5,11,12],
(2) The extent to which, at an individual level, strengths and weaknesses in individual disciplines or clusters of disciplines can be balanced and/or overcompensated.

We will examine the extent to which the various strengths of the athletes are favored by the predefined disciplinary structure of the heptathlon [13]. Although the structure of the heptathlon has been created to implicitly consider a well-balanced athlete type [2], athletes with fast-twitch muscle fibers seem to be at an advantage in some disciplines. For example, high performance in speed, explosive strength, and speed strength is mainly associated with a high performance in jump, throw, put, and sprint disciplines, whereas aerobic or anaerobic, endurance-focused factors with a higher share of slow-twitch muscle fibers take weight only in the 800 m run. In this context, Vindusková [14] noted that the heptathlon is a technical discipline with strength and speed character for which “maximum speed”, “explosive power”, and “speed endurance” are the key performance factors.

## 2. Methodology

### 2.1. Sampling and Survey Procedures

Data analysis was based on the data recorded for the individual performances of the 10 winning heptathletes in the World Athletics Championships from 1987 to 2013 and the Olympic Games from 1988 to 2012. Previous results were not included because the current conversion formula has been applied only since 1985 [1]. For each athlete, ranking; name; nationality; date of birth; year of competition; results achieved in seconds, centimeters, and meters; points calculated for each discipline; and the total number of points were determined. Data sources included heptathlon results published in “Leichtathletik” magazine, on the webpage “Sports Reference—Sports Statistics Quickly, Easily & Accurately”, and on the official webpage of the International Association of Athletics Federations (IAAF). Comparing the contents of the various media ensured data consistency. Additional specific Internet research on spelling and change of names, dates of births, *etc.*, resulted in a comprehensive list of athletes comprising 200 consistent data records (consisting of 13 datasets for world championships and 7 datasets for Olympic Games, with 10 athletes in each case).

### 2.2. Data Analysis

**Figure 1:**
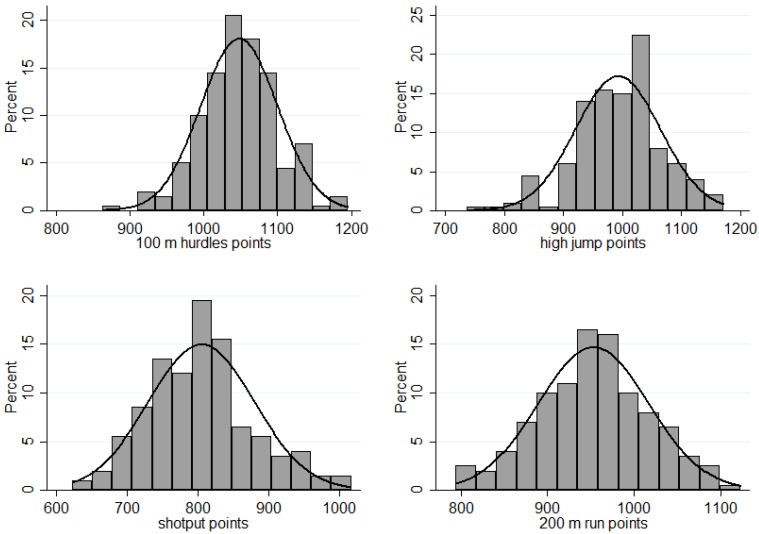

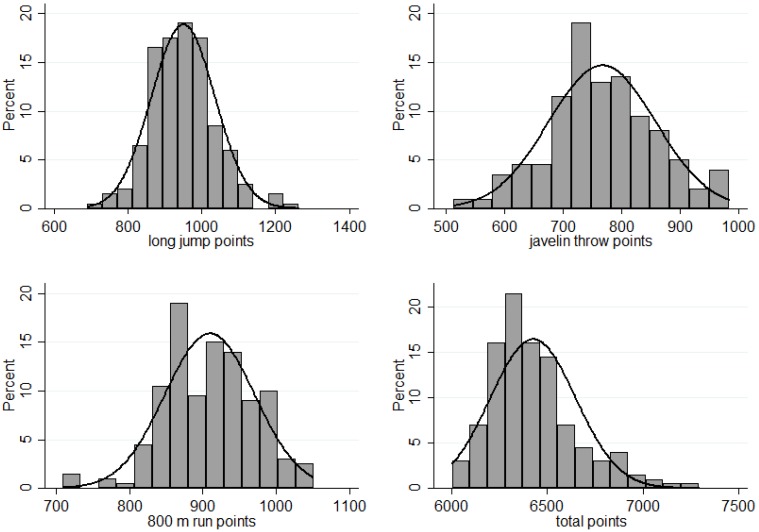
Distribution of the achieved points (n = 200).

The software program used for data analysis was Stata 12. In addition to descriptive analysis methods (e.g., mean values, standard deviation, and frequencies), correlations, bivariate and multivariate linear regressions, and panel data regressions were applied to investigate the research questions mentioned above. Kihlberg and Karvonen (1957) showed that the distributions of the best performances are formally similar to the Pareto and not to the Normal distribution [15]. However the distributions of achieved points in heptathlon resemble a Normal more than a Pareto distribution (Figure 1). Therefore it is possible to compute usual descriptive and inferential statistics.

To examine the influence of individual performance on the number of points achieved in each discipline, the relevant values needed to be standardized because individual performances are measured in their respective units, and it is naturally impossible to directly compare times, distances, or heights. Therefore, all performances were standardized, or z-transformed. 

To analyze the impact of standardized performance on points achieved, seven bivariate linear regressions were estimated and used as a basis for the calculation of marginal effects [16]. Because the curves of the power functions increase positively, sub-disciplines should explain the total amount of variance. For example, to verify that high performance in speed, explosive strength, and speed strength is mainly associated with high performance in jump, throw, put, and sprint disciplines, whereas endurance-focused factors take weight only in the 800 m run, the number of points in the individual disciplines was correlated with the number of total points achieved by means of linear regression, and correlation calculations between the individual disciplines were performed. Owing to potential correlations between performances in the same competitions, we performed seven linear regressions with adjusted standard error for every Olympic Games or world championship.

To obtain additional information on sub-discipline association other than from the correlation matrix, a main component analysis with orthogonalization was performed [17]. “The central idea of principal component analysis is to reduce the dimensionality of a data set in which there are a large number of interrelated variables, while retaining as much as possible of the variation present in the data set” [17]. Through the analysis, a new set of variables is created, including almost all information on the original variables. This factor analysis enables a summary of sub-disciplines. We performed a main component factor analysis with orthogonal varimax rotation. Based on the scree plot of the factor analysis (eigenvalue diagram) and the elbow criterion, the following two implied factors follow: the eigenvalue with two factors is 1.34, compared to 0.98 for a three-factor solution (see Table 1).

The disciplines were divided into two factors as follows:

- Factor 1 *"*Speed*"*: 100 m hurdles, 200 m run, long jump, high jump;
- Factor 2 “Strength”: Javelin throw and shotput.

The final 800 m run was not assigned to any factor. Taking the eigenvalue criterion as a basis, a three-factor solution follows with factor 1 comprising 100 m hurdles, 200 m run, and long jump, factor 2 including shotput and javelin throw, and factor 3 consisting of high jump. The 800 m run was again not assigned to any factor (Karlis *et al.*, 2003; Letzelter, 1985). 

Because the high jump is primarily determined by speed, explosive strength, and speed strength from a training-scientific point of view and thus correlates much more intensely with the sprint disciplines, we decided to employ the first variant with the high jump being assigned to factor 1.

We performed three multiple linear regressions with each factor, represented through corresponding variables to examine the impact of factors on the total number of points. We used regressions with the cluster option again to indicate that the observations were clustered into the 20 competitions, owing to potential correlations between performances in the same competitions.

In addition, a cluster analysis was performed to examine whether athletes exist with systematic similarities to other athletes in terms of points achieved [18]. “Cluster analysis is an exploratory data analysis tool for organizing observed data into meaningful taxonomies, groups, or clusters, based on combinations of IV’s (instrumental variables), which maximizes the similarity of cases within each cluster while maximizing the dissimilarity between groups…” [19]. We used Ward's method to get an impression of the possible number of clusters, reviewed the dendrogram and performed the K-means cluster analysis [19]). The structure of the heptathlon implicitly applies the athletes' versatility, which is intended to reflect the generalist of athletics (the supreme discipline in women's athletics). Therefore, we aim to verify whether the requirements to live up to this claim are met or athletes specialized in individual disciplines or groups of disciplines have an advantage over generalists.

By means of a cluster analysis using a dendrogram, a two-cluster solution for generalists and specialists has been identified. The level of significance and thus the forecast value were specified to be 5% for all test procedures. The pertinent effect sizes were calculated for the explanatory value.

## 3. Results

### 3.1. Performance in Sub-Discipline and Points Achieved in Sub-Disciplines

By means of bivariate regression calculations—based on standardized performances—we could show that athletes attaining javelin throw, long jump, and shotput perform better than one, two, or three standard deviations compared to the average results achieve the highest increase in points (slope coefficient) in relative terms. In contrast, the relative increase in the disciplines of the 200 m and 800 m runs, high jump, and 100 m hurdles is significantly lower. For example, a higher javelin throw result by one standard deviation can lead to a relative performance improvement of 12.5%, whereas an increase by one standard deviation in the 100 m hurdles involves a performance improvement of only approximately 5%. Nevertheless, the analysis in Table 2 shows that a higher javelin throw result by three standard deviations (1047.1 points) corresponds to a merely average performance in 100 m hurdles (1047.3 points).

On the basis of the constants, it can also be deduced that the single disciplines on the one hand each contribute differently to the overall result [2], which, for example, becomes strongly evident in positive terms for 100 m hurdles and in negative terms for the javelin throw. However, for the high and long jump, the constants are already quite distinct, an effect that is even more intensified through high slope coefficients.

As shown in Table 2, the slope coefficients of the sub-disciplines of javelin throw, long jump, shotput, and high jump are the highest, whereas lesser coherences are observed in the 200 m run, 800 m run, and 100 hurdles. Therefore, the initial assumption is that athletes with a high performance in these disciplines are rewarded with a high number of points. This is, however, absolutely not the case (with the exception of javelin throw and shotput with their high slope coefficient but low constant) because the average number of points achieved in the disciplines of shotput, javelin throw, and 800 m run is below the number of points achieved in the other disciplines; thus, a performance efficiency that is higher in relative terms takes effect here. At the same time, the athletes achieve a significantly lower number of points owing to the low constant in these sub-disciplines. 

### 3.2. Points Achieved in Individual Disciplines and Total Number of Points

Table 3 and Table 4 illustrate that all sub-disciplines have a highly significant impact on the final result but with varying explanatory power, which is explained by the different correlation of the points achieved within the sub-disciplines. The strongest influence was evident for the sub-disciplines of long jump, 200 m run, 100 m hurdles, and high jump with a variance clarification of 48% to 21%. The high degree of explanatory power of up to 48% in the case of the long jump is due to the inter-correlation with additional performance factors, such as starting speed (100 m hurdles and 200 m run) and jump impulse (see correlation coefficient of r = 0.32 (p < 0.001) between the high and long jumps) [5,9,20]. Shotput, javelin throw, and the final 800 m run are also significantly associated with the final result, but they can be deemed marginal in their explanatory power based on the R^2^ values (variance clarification 7% to 8%). 

When looking at the sub-disciplines, significant and moderate correlations can be observed between the 100 m hurdles and 200 m run (0.61), 100 m hurdles and long jump (0.45), high jump and long jump (0.32), 200 m run and long jump (0.48), and 200 m and 800 m runs (0.41) [21]. The javelin throw and 800 m run have a negative correlation (−0.35).

Analyzing the inter-correlation of the disciplines, it becomes evident that a specific type of athlete frequently achieves good performance in these sub-disciplines. Phenotypically, this type shows distinct strengths in the sprint jump area. To develop a better understanding of this information, a factor analysis will examine the extent to which discipline groups can be identified in the heptathlon and which characteristics they carry [5,7,9].

### 3.3. Factor-Analytic Structure of Heptathlon and Total Number of Points

The following three basic discipline groups are the result of a main component analysis: the “Speed” type included 100 m hurdles, high jump, 200 m run, and long jump. The “Strength” type (maximum strength and speed strength) comprised shotput and javelin throw, and the “Endurance” type consisted of the 800 m run [5].

To examine the impact of factors on the total number of points, we performed three multiple linear regressions with each factor represented by the corresponding variables (Table 5). The “Speed” model with the 100 m hurdles, high jump, 200 m run, and long jump explained 72% of variances (R²), whereas the “Strength” model explained only 10% and the “Endurance” model 7%. However, given that these regression models consisted of different numbers of variables, we calculated average values by dividing R² by the number of independent variables. Again, the “Speed” model explained the largest amount of variance, followed by the “Endurance” and “Strength” models.

### 3.4. Cluster-Analytic Structure of the Heptathlon Type and Achieved Total Number of Points

**Figure 2:**
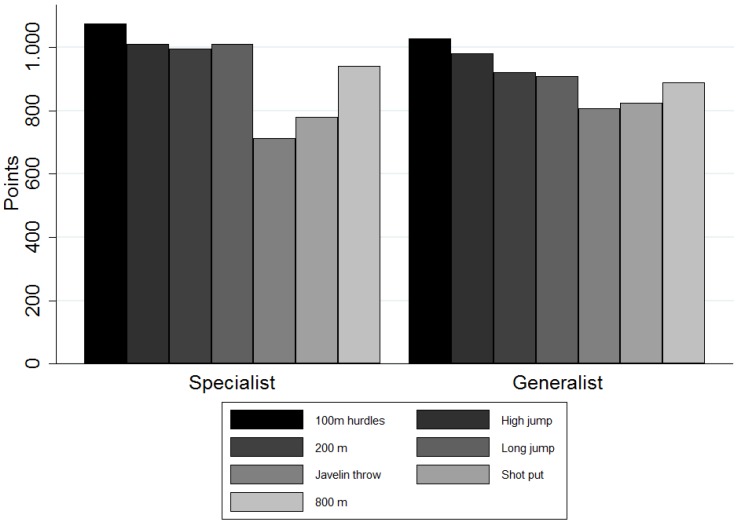
Points achieved by specialists and generalists in the individual disciplines by means of a two-cluster solution.

The generalists achieved a mean number of points (±SD) of 6349 ± 166 (n = 109), whereas the specialists reached 6518 ± 246 points (n = 85). The generalists' share in the sample was 56% *versus* a 44% share of the specialists (see Figure 2). Six heptathletes could not be assigned to any type in the sample.

The sequencing of the analysis of type changes between participations of an athlete in various competitions led to the conclusion that athletes who reached a ranking among the top 10 in Olympic Games or world championships usually abide by their type or do not want to change it. This means that an athlete diagnosed as a specialist was also identified as such in the subsequent competitions. Of the 82 athletes who participated in a total of 194 competitions, only 11 changed their type and 33 stuck by their type during that time.

## 4. Discussion

The structural analysis of the women’s heptathlon yielded empirical evidence that in an allegedly objectively quantifiable form of such sports as the women’s heptathlon, the normatively influenced transformation of performance into points using a conversion formula—applied to the ratios between speed disciplines (sprints and jumps), strength requirements (throw and put disciplines) and endurance (800 m)—results in an imbalanced weighting of the performances in the seven disciplines and thus impacts the final result in different ways [4]. This result agrees almost completely with the findings identified for the men's decathlon [6,7,20,22].

Currently, heptathlon performance is disproportionately influenced by a high performance in 100 m hurdles and the performances in the high and long jumps, whereas javelin throw performance is underrepresented in the overall competition performance and thus probably bears the highest training-specific development potential. The long jump in the women’s heptathlon is of special importance, as reflected in the high variance clarification of 48% and explainable by the high degree of inter-correlation with the disciplines of 100 m hurdles, high jump, and 200 m run. Physiologically, this can be explained by the high degree of speed, explosive strength, and speed strength in this discipline group, which the cluster analysis with a two-factor solution confirms phenotypically as the sprint-jump type. By comparison, Park and Zatsiorsky [20] found a 43.1% variance clarification in the men’s decathlon for the cluster consisting of 100 m run, 400 m run, 110 m hurdles, and long jump, interpreted by the authors as “sprinting performance” [8,10].

The higher shares of maximum strength requirements were attributed to the second factor and were expressed in the disciplines of javelin throw and shotput. The final 800 m as the alleged endurance part in the heptathlon could not be assigned to any factor because, as Letzelter [5] already postulated, it is … “not long enough to check endurance exclusively”. (Schomaker & Heumann, 2011; Wimmer *et al.*, 2011) used men’s decathlon results to identify a three-factor solution comprising the factors “speed-and-athletic” (100 m, long jump, 400 m, and 110 m hurdles), “strength-and-technique” (shotput, high jump, and discus throw), and “endurance” as a special factor with the final 1500 m run, which largely corresponds to the data presented for the heptathlon.

The following should therefore be considered: (a) the extent to which the dominance of the speed and strength shares could be changed for the benefit of the endurance shares (achieving a balance of the motor capabilities endurance, strength, and speed), and (b) the extent to which the internal respective discipline structures unambiguously represent the basic motor capabilities. To develop these two ideas, further discussion is needed regarding whether the introduction of hammer throw as a more strength-determined discipline to replace javelin throw or the extent to which a final 3000 m or 5000 m run might be more suitable for the representation and weighting of strength with respect to endurance than the 800 m run. These amendments might result in a higher balance within the individual heptathlon disciplines. Implicitly, the specialist in versatility is considered the model athlete in the context of the women’s heptathlon, which is expressed, for example, in the German term “Königin der Athletinnen” (queen of female athletes) alluding to the heptathlon as the supreme discipline in women's athletics. Nevertheless, the extent to which historically changed framework conditions, specific selection and support mechanisms, device-based innovations, and the 30-year-old transformation rule influence the chances of even a real specialist reaching a higher performance and thus a higher probability to win is a matter of debate [7,13]. The cluster analysis could show for the present examination sample, pertaining to the participants in world championships and Olympic Games, that the specialists achieved an average of approximately 170 points more than the generalists and that specialists are found among the world's top athletes approximately 12% more often than generalists, which would explain early selection mechanisms based on good sprint-jump performances. Furthermore, the generalists are relatively more time-stable than the specialists, and the typology applied remained consistent during the competitions. 

Analogous to the results identified for the men's decathlon [6], and if equally weighted disciplines are the goal, the present results for the heptathlon show the necessity of a structural reevaluation of the points formula and/or an extension of the disciplines (decathlon or dodecathlon) because the now 30-year-old transformation of performance in the sub-disciplines leads to distortions in the overall result [1]. Thus, in the sense of equality, the women’s decathlon instead of heptathlon might be conceivable as an official discipline in women’s all-rounder competitions.

## Figures and Tables

**Table 1:** Results of the exploratory factor analysis via orthogonal rotation, factor loadings (n = 200).

Disziplines	Factor 1	Factor 2
100 m hurdles	0.73	−0.24
High jump	0.50	0.37
200 m run	0.75	−0.39
Long jump	0.83	0.06
Shotput	−0.07	0.54
Javelin throw	−0.07	0.70
800 m run	0.24	−0.73

**Table 2:** Results of the bivariate linear regressions of the standardized performances on the respective points achieved in the individual disciplines (n = 200).

Individual Discipline	Slope Coefficient	Constant	Marginal Effect		
			(+1 STD)	(+2 STD)	(+3 STD)
100 m hurdles	52.16 ***	1047.3 ***	1099.4	1151.6	1203.8
High jump	71.41 ***	993.7 ***	1065.1	1136.5	1207.9
Shot put	75.73 ***	800.9 ***	876.6	952.4	1028.1
200 m run	63.63 ***	952.0 ***	1015.7	1079.3	1142.9
Long jump	86.66 ***	949.3 ***	1036.0	1122.6	1209.3
Javelin throw	95.23 ***	761.6 ***	856.8	952.0	1047.1
800 m run	60.52 ***	909.8 ***	970.4	1030.9	1091.4

*** p < 0.001.

**Table 3:** Results of the bivariate linear regressions of the rounded number of points achieved in the individual disciplines on the total number of points (n = 200).

Individual Discipline	Slope Coefficient	Constant	R²
100 m hurdles	2.479 ***	3827.1 ***	0.333
High jump	1.533 ***	4903.4 ***	0.208
Shot put	0.705 **	5858.1 ***	0.075
200 m run	2.141 ***	4384.7 ***	0.380
Long jump	1.818 ***	4698.3 ***	0.480
Javelin throw	0.629 ***	5943.1 ***	0.078
800 m run	0.958 **	5553.8 ***	0.069

** p < 0.01, *** p < 0.001.

**Table 4:** Correlation matrix of the points achieved in the individual disciplines and the total number of points (significance test in parentheses).

	TP	100	HI	SP	200	LS	JT	800
TP	1							
100	**0.58**	1						
	(0.00)							
HI	**0.49**	0.04	1					
	(0.00)	(0.59)						
SP	0.24	−0.14	−0.06	1				
	(0.00)	(0.04)	(0.44)					
200	**0.61**	**0.61**	0.10	−0.15	1			
	(0.00)	(0.00)	(0.14)	(0.04)				
LS	**0.70**)	**0.45**	**0.32**	−0.03	**0.48**	1		
	(0.00	(0.00)	(0.00)	(0.64)	(0.00)			
JS	0.26	−0.09	0.07	0.19	−0.24	−0.13	1	
	(0.00)	(0.18)	(0.31)	(0.01)	(0.00)	(0.06)		
800	0.26	0.25	−0.01	−0.26	**0.41**	0.12	−**0.35**	1
	(0.00)	(0.00)	(0.89)	(0.00)	(0.00)	(0.09)	(0.00)	

TP = Total points, 100 = 100 m hurdles, HI = high jump, SP = shotput, 200 = 200 m run, LS = long jump, SW = javelin throw, 800 = 800 m run, correlation ≥ 0.30 are in bold; according to Dancey and Reidy’s (2004, p. 171) these are weak correlations.

**Table 5:** Results of the multivariate linear regressions of the rounded number of points achieved in the individual disciplines or factors on the total number of points (n = 200).

Disciplines	Model 1 Factor 1 (Speed)	Model 2 Factor 2 (Strength)	Model 3 Factor 3 (Endurance)
100 m hurdles	1.077 *** (4.60)		
High jump	1.064 *** (11.16)		
200 m run	0.872 *** (5.94)		
Long jump	0.925 *** (7.05)		
Shot put		0.578 * (2.54)	
Javelin throw		0.537 *** (4.50)	
800 m run			0.958 ** (3.22)
Constant	2531.1 *** (11.88)	5548.0 *** (30.37)	5553.8 *** (20.64)
N	200	200	200
R^2^	0.721	0.102	0.069
Adjusted R^2^	0.715	0.093	0.065
Average R² (R²/number of IV)	0.180	0.051	0.069

Regression coefficients and t statistics in parentheses are displayed + p < 0.10, * p < 0.05, ** p < 0.01, *** p < 0.001.

## References

[1] Trkal V. (2003). The development of combined events scoring tables and implications for the training of decathletes. New Stud. Athl..

[2] Westera W. Under Attack: The Heptathlon Scoring Method. http://www.athleticscoaching.ca.

[3] Pitsch W., Emrich E., Fröhlich M., Flatau J. (2006). Zur Legitimation von Normen im Sport am Beispiel des Mehrkampfs in der Leichtathletik—Rechtsphilosophische und rechtssoziologische Positionen. Leipz. Sportwiss. Beitr..

[4] Cox T.F., Dunn R.T. (2002). An analysis of decathlon data. J. R. Stat. Soc. Ser. D.

[5] Letzelter M., Müller N., Augustin D., Hunger B. (1985). Zur Struktur des Siebenkampfes: Einflusshöhe und interne Verwandtschaft der Einzelübungen. Frauenleichtathletik.

[6] Westera W. (2006). Decathlon, towards a balanced and sustainable performance assessment method. New Stud. Athl. IAAF.

[7] Kenny I.C., Sprevak D., Sharp C., Boreham C. (2005). Determinants of success in the olympic decathlon: Some statistical evidence. J. Quant. Anal. Sports.

[8] Schomaker M., Heumann C. (2011). Model averaging in factor analysis: An analysis of olympic decathlon data. J. Quant. Anal. Sports.

[9] Woolf A., Ansley L., Bidgood P. (2007). Grouping of decathlon disciplines. J. Quant. Anal. Sports.

[10] Wimmer V., Fenske N., Pyrka P., Fahrmeir L. (2011). Exploring competition performance in decathlon using semi-parametric latent variable models. J. Quant. Anal. Sports.

[11] Karlis D., Saporta G., Spinakis A. (2003). A simple rule for the selection of principal components. Commun. Stat. Theory Methods.

[12] Dawkins B.P., Andreae P.M., O’Conner P.M. (1994). Analysis of olympic heptathlon data. J. Am. Stat. Assoc..

[13] Van Damme R., Wilson R.S., Vanhooydonck B., Aerts P. (2002). Performance constraints in decathletes. Nature.

[14] Vindusková J. (2003). Training women for the heptathlon—A brief outline. New Stud. Athl..

[15] Kihlberga J., Karvonena M.J. (1957). Comparison on statistical basis of achievement in track and field events. Res. Q. Am. Assoc. Health Phys. Educ. Recreat..

[16] Greene W.H. (2008). Econometric Analysis.

[17] Jolliffe I.T. (2002). Principal Component Analysis.

[18] Wiedenbeck M., Züll C., Wolf C., Best H. (2010). Clusterananlyse. Handbuch der Sozialwissenschaftlichen Datenanalyse.

[19] Burns R.P., Burns R. (2008). Business Research Methods and Statistics Using SPSS.

[20] Park J., Zatsiorsky V.M. (2011). Multivariate statistical analysis of decathlon performance results in olympic athletes (1988–2008). World Acad. Sci. Eng. Technol..

[21] Fanshawe T. (2012). Seven into two: Principal components analysis and the olympic heptathlon. Significance.

[22] Linden M. (1977). Factor analytical study of olympic decathlon data. Res. Q. Am. Alliance Health Phys. Educ. Recreat..

